# Fast verification of Gamma Knife™ treatment plans

**DOI:** 10.1120/jacmp.v1i4.2638

**Published:** 2000-09-01

**Authors:** Pengpeng Zhang, David Dean, Q. Jackie Wu, Claudio Sibata

**Affiliations:** ^1^ Department of Biomedical Engineering Case Western Reserve University Cleveland Ohio 44106; ^2^ Department of Neurological Surgery University Hospitals of Cleveland/Case Western Reserve University Cleveland Ohio 44106; ^3^ Department of Radiation Oncology University Hospitals of Cleveland/Case Western Reserve University Cleveland Ohio 44106

**Keywords:** second check, Gamma Knife™ radiosurgery, irradiation time

## Abstract

The Leksell stereotactic Gamma Knife™ uses radiation from 201 C60o sources that are focused to the center of a collimator helmet to deliver a high dose of radiation with minimal irradiation of proximal structures. This paper presents a method for fast verification of the irradiation time as calculated by the Leksell Gamma Knife™ treatment planning software GammaPlan®. To obtain the irradiation time for each shot in the treatment plan, one must first accurately calculate the tissue maximum ratio (TMR) for each of the individual 201 beams. The algorithm presented in this paper begins with the determination of the geometrical relationship between the Gamma Knife™ collimator helmet and the stereotactic frame. A group of reference points is measured to build a head model simulating the patient skull geometry. During radiosurgery, the isocenter of the collimator helmet is moved to the shot center. A group of spatial vectors describing the reference points at the skull surface is obtained by converting the Cartesian coordinates to Polar coordinates. For each individual beam, the three nearest reference vectors are found by ranking the relative angles. The depth that each beam penetrates the patient's skull to the isocenter is obtained via linear interpolation. The TMR for each beam then is compared with the TMR for the calibration setup, which is done using a spherical 8 cm radius phantom. This algorithm is applied to verify the treatment time calculated in GammaPlan® Version 5.2. The results are shown to agree with GammaPlan® within 3%.

PACS number(s): 87.52.–g, 87.66.–a

## I. INTRODUCTION

It is beneficial to have a second calculation to verify the treatment planning system, thereby detecting potential dosimetry errors. Traditionally, a therapy plan is checked at one or more computation points via manual calculation. For Gamma Knife™ stereotactic radiosurgery such a check is not possible due to the complex patient and source geometry. We are not aware of the existence of any Gamma Knife™ second check systems. The goal of this work is to fill this void.

We present a program that verifies radiation shot delivery time of Gamma Knife™ treatment plans. The Leksell stereotactic Gamma Knife™ uses radiation from 201 Co60 sources that are focused on the center of a collimator helmet. These beams create an oval shape dose distribution at the helmet center. Each beam focuses on the isocenter, which is 40 cm from each source. The dose rate at the isocenter of each beam is the product of four factors: the dose rate determined at the calibration time, the time decay factor, the collimator factor, and exponential attenuation through the skull.^1^ ^,^ ^2^ The total dose rate at the isocenter is the sum of individual beam dose rates. These four factors are easy to derive except for the attenuation factor, which depends on the depth of each beam and requires knowledge of the geometry setup of the collimator helmet and the patient's skull. To obtain the needed geometrical information, as well as to provide rigid skull fixation, the collimator helmet is used in conjunction with a stereotactic frame attached by four pins to the outer surface of the patient skull.^3^ ^,^ ^4^ Twenty‐four preselected points on the skull are measured to construct a spatial model simulating the three‐dimensional (3D) relationship between the patient's skull and the collimator helmet. Based on this model, we propose an algorithm that is able to verify the irradiation time produced by the treatment planning software, GammaPlan®. In this paper, we note that all results were compared with the Leksell GammaPlan® Version 5.2 running on the Leksell Gamma Knife™ unit model *B* at the University Hospitals of Cleveland.

## II. METHODS

### A. Gamma Knife™ collimator setup geometry

**Figure 1:**
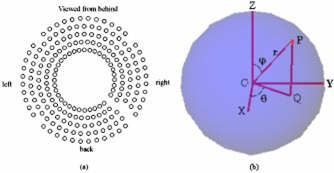
Illustration of the Gamma Knife™ setup. (a) 201 collimators are divided into five layers. (b) (Color) Coordinate system.

On each collimator helmet, the 201 collimators can be divided into five layers as illustrated in Fig. 1(a)). Each layer has the same azimuth angle, and each collimator in the same layer is separated from its neighbors with the same angle interval as described in Table I. One collimator in layers 1 to 4 and 4 collimators in layer 5 are not punched during manufacturing. By default, the weight for each collimator is set to 1. Asymmetric dose patterns are obtained by blocking given collimators via setting the corresponding weight to zero. The spatial direction of each radiation beam from each collimator is represented in Fig. 1(b), where *O* is the isocenter, *OP* is the radiation beam, φ is the azimuth angle, and θ is the plane angle (<QOX).

**Table I:** Gamma knife™ collimators setup.

Layer	No. Collimators	Interval degree α	φ (azimuth angle)
1	35(1)	10°	55.7°
2	39(1)	9°	63.9°
3	39(1)	9°	71.8°
4	44(1)	8°	79.2°
5	44(4)	7.5°	86.4°

### B. Patient skull geometry model

**Figure 2:**
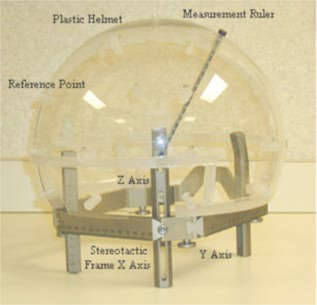
(Color) A plastic helmet is mounted on the patient's stereotactic frame before surgery. A ruler is used to measure the distance between the helmet surface and the pateint's scalp at 24 reference points.

Before radiosurgery, a stereotactic frame is mounted on the patient's head as shown in Fig. 2. The distances from the center of the stereotactic frame to 24 preselected points on patient's scalp are measured with a special plastic helmet attached to the frame. The 24 points can be divided into four layers; each has a different azimuth angle and the same angle interval. The spatial setup of the 24 points is shown in Table II.

**Table II:** Patient's plastic helmet setup.

Layer	Interval degree α	φ (azimuth angle)
*A*	45°	37.5°
*B*	45°	63.8°
*C*	45°	90°
*D*	45°	107.6°

Based on the points measured on the skull, a 3D model simulating the geometry of the skull surface inside the stereotactic frame is created by GammaPlan® (Leksell Gamma Knife™ Unit User's Manual Vol. 2, Elekta, Norcross, GA). Thirty‐two reference points in the model defining the patient's skull geometry are shown in Table III, where bold numbers are real measurements, and light‐faced numbers are obtained from interpolation.

**Table III:** Skull geometry determined by 32 reference points. Twenty‐four (in bold type) are actual. Eight are estimated.

	1	2	3	4	5	6	7	8
*A*	**88.0**	90.8	**97.0**	92.7	**90.0**	91.6	**98.0**	93.5
*B*	84.6	**92.0**	100.2	**95.0**	87.3	**93.0**	99.1	**97.0**
*C*	**82.0**	**93.0**	**101.0**	**90.0**	**85.0**	**91.0**	**100.0**	**95.0**
*D*	**82.0**	**93.0**	95.8	**95.0**	**85.0**	**99.0**	**105.0**	**96.0**

### C. Calculation of the beam depth

The key point in calculating the time to deliver each shot is to obtain the depth, $D_{j}$, each beam penetrates the skull to reach the helmet center. The stereotactic frame each patient wears has the coordinate system shown in Fig. 2. When measuring the reference points on the scalp, the center of the plastic helmet has the coordinate of (100, 100, 100) inside the stereotactic frame. Just before delivering each shot, by adjusting the relative position between the collimator helmet and the stereotactic frame, the isocenter of the shot is transferred to the isocenter of collimator helmet. After this setup, the coordinates of reference points listed in Table III also translate to their new coordinates, which can be obtained using Eqs. (1), (2), and (3):

(1) $$X_{m}^{\prime }=X_{m}+X_{s}-X_{0},$$

(2) $$Y_{m}^{\prime }=Y_{m}+Y_{s}-Y_{0},$$

(3) $$Z_{m}^{\prime }=Z_{m}+Z_{s}-Z_{0},$$

where $X_{s},Y_{s}$, and $Z_{s}$ are the shot center coordinates inside the stereotactic frame system, $X_{0},Y_{0}$, and $Z_{0}$ are the stereotactic frame center [i.e., (100, 100, 100)], and $X_{m}^{\prime },Y_{m}^{\prime }$, and $Z_{m}^{\prime }$ are the coordinates after the translocation.

GammaPlan® obtains $D_{j}$ for each beam *j* in a Cartesian coordinate using a surface spline. In this paper, we present another fast method to obtain $D_{j}$. First, the Cartesian coordinate of each reference point is converted to its Polar coordinate, with the origin at the isocenter, as described by Eqs. (4) and (5),

(4) $$V_{m}=X_{m}^{\prime }\vec{i}+Y_{m}^{\prime }\vec{j}+Z_{m}^{\prime }\vec{k},$$

(5) $$|V_{m}|=\sqrt{{\left(X_{m}^{\prime }\right)}^{2}+{\left(Y_{m}^{\prime }\right)}^{2}+{\left(Z_{m}^{\prime }\right)}^{2}},$$

where $V_{m}$ is the vector representing the reference point in the Polar coordinate and $\left|V_{m}\right|$ is its length. Then, a spatial vector, $V_{c}$, as described by Eq. (6), represents each radiation beam,

(6) $$V_{c}=\cos(\varphi )\cos(\theta )\vec{i}+\cos(\varphi )\sin(\theta )\vec{j}+\sin(\varphi )\vec{k},$$

where φ is the azimuth angle and θ is the plane angle. Thus the spatial angle between each beam vector and the reference point vector is obtained from Eq. (7),

(7) $$\beta =\cos^{-1}\left(\frac{V_{m}^{\prime }V_{c}}{\left|V_{m}^{\prime }|\times |V_{c}\right|}\right).$$

Three nearest reference points to each beam are selected by sorting the spatial angle β. Finally, a linear interpolation is used in Eq. (8) to obtain the depth, $D_{j}$ for each beam,

(8) $$D_{j}=\frac{\sum_{i=1}^{3}R_{i}/\theta_{i}}{\sum_{i=1}^{3}1/\theta_{i}}.$$

### D. Time calculation for each shot

**Figure 3:**
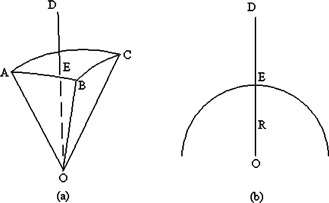
Single beam geometry. (a) Treatment setup. (b) Calibration setup.

During radiosurgery, the spatial relationship between a beam and the patient's skull surface is illustrated in Fig. 3(a), where *A, B*, and *C* are reference points on the skull surface, *O* is the isocenter, line *DO* is the path of the beam, *E* is the intersection point between *DO* and surface *ABC*, and segment *OE* is the depth the beam penetrates the skull to reach the center, i.e., $D_{j}$. The setup of the calibration with a 80‐mm radius sphere phantom is illustrated in Fig. 3(b), where *O* is the isocenter, *DO* is the path of the beam, and *DO* is the reference depth of the calibration phantom. Given this setup, for each beam *j*, ${\text{TMR}}_{j}$ can be obtained as

(9) $${\text{TMR}}_{j}=e^{-\mu (D_{j}-D_{0})}$$

where μ is the attenuation coefficient for C60o(0.0063 mm−1). Then the dose rate at isocenter, ${\text{DR}}_{\text{isocenter}}$, can be obtained as

(10) $${\text{DR}}_{\text{isocenter}}=\sum_{j=1}^{201}{\text{TMR}}_{j}\times W_{j}\times {\text{CF}}_{h}\times {\text{DR}}_{0}\times e^{-t/T_{1/2}},$$

where ${\text{DR}}_{0}$ is the calibration dose rate at the center of the 18‐mm collimator helmet with the calibration phantom, $W_{j}$ is the weight for each beam (default value is 1), ${\text{CF}}_{h}$ is the collimators factor for each collimator helmet, *t* is the time span between treatment and calibration, and *T* is the half‐life of C60o (5.26 years). Finally, the time to deliver each shot, ${\text{Time}}_{s}$, is

(11) $${\text{Time}}_{s}=\text{DP}\times \text{NF}\times W_{s}/{\text{DR}}_{\text{isocenter}},$$

where DP is the prescribed radiation dose, $W_{s}$ is the weight for the shot, and NF is a normalization factor.

## III. RESULTS AND DISCUSSION

### A. Accuracy of the treatment time calculation

An ellipsoid phantom was simulated to test the accuracy of calculating the depths and treatment time using our algorithm. This phantom can be described by Eq. (12), where we use a=0.95,b=0.9, and R=80, to simulate a head,

(12) $${\left(X-100\right)}^{2}+a\times {\left(Y-100\right)}^{2}+b\times {\left(Z-100\right)}^{2}=R^{2}.$$

First, the center of the ellipsoid phantom is placed at the isocenter of the helmet. Because the geometry of the phantom is well defined, the distances between the 32 reference points and the isocenter can be calculated. Given this information, a 3D head model can be created. Then during radiosurgery, the isocenter of the helmet is moved to the center of the shot. In the new coordinate frame, the phantom can be described by Eq. (13), where we use $X_{s}=125,\;Y_{s}=125$, and $Z_{s}=125$,

(13) $${\left(X-X_{s}\right)}^{2}+a\times {\left(Y-Y_{s}\right)}^{2}+b\times {\left(Z-Z_{s}\right)}^{2}=R^{2}.$$

Given the full knowledge of the system's geometric setup, we can calculate each 201$D_{j}$ depth. Meanwhile, each $D_{j}$ can also be obtained by using the head model defined via the 32 reference points.

Given Eqs. (9), (10), and (11), following the derivative chain rule, we obtain:

(14) $$\frac{\Delta \text{Time}}{\text{Time}}=-\frac{\Delta {\text{DR}}_{\text{isocenter}}}{{\text{DR}}_{\text{isocenter}}}=\mu \times \sum_{j=1}^{201}\Delta D_{j}.$$

Plug in the theoretical 201 $D_{j}$, and the 201 $D_{j}$ obtained by using the head model, the relative error between the two methods is less than 0.66%. Thus, using the method presented in this paper to calculate the treatment time does not produce significant relative error to the treatment time.

### B. Comparison with GammaPlan®

Two examples comparing the method presented in this paper with the GammaPlan® 5.2 are shown in Tables IV and V where Time I and Time II represent the treatment time obtained from GammaPlan® and the presented method, respectively. The presented method agrees with GammaPlan® within 3%.

**Table IV:** A Leksell gamma knife (LGK) plan with one shot.

Shot no.	*X* (mm)	*Y* (mm)	*Z* (mm)	Collimator	Weight	Time I (min)	Time II (min)	Difference
1	98.0	153.5	69.5	8	1.0	12.93	13.05	0.9%

**Table V:** An LGK plan with multiple shots.

Shot no.	*X* (mm)	*Y* (mm)	*Z* (mm)	Collimator	Weight	Time I (min)	Time II (min)	Difference
1	101.5	84.0	139.5	8	1.20	3.59	3.61	0.6%
2	105.5	92.5	144.0	8	1.15	3.45	3.50	1.4%
3	100.5	82.0	144.5	8	1.15	3.44	3.47	0.9%
4	106.0	84.0	149.0	8	1.00	2.98	3.04	2.0%
5	106.0	85.0	142.5	4	1.40	4.97	5.06	1.6%
6	100.0	88.0	144.5	4	1.30	4.69	4.71	0.4%
7	114.0	88.5	141.0	4	1.30	4.58	4.71	2.8%
8	101.0	88.0	148.0	4	1.15	4.14	4.20	1.4%
9	109.0	89.5	140.0	4	1.05	3.72	3.79	1.9%
10	102.0	90.0	140.5	4	1.00	3.59	3.61	0.6%
11	102.0	84.0	138.0	4	1.00	3.57	3.58	0.3%
12	103.0	88.5	137.5	4	0.90	3.22	3.23	0.3%

In GammaPlan® the patient's skull is modeled as a group of patches via a surface spline of the reference points. Thus the intersection point of each radiation beam meeting the skull surface is obtained as the beam crosses the corresponding patch. The method proposed in this paper simplifies the procedure via translation from Cartesian coordinate to Polar coordinate and linear interpolation of the nearest vectors. For a single‐shot treatment plan, the presented method agrees with GammaPlan® within 1%. For a multiple‐shot treatment plan, the relative error is higher because the error for each beam accumulates to affect the normalization factor used in Eq. (11).

The presented algorithm is implemented in MATLAB™ (MathWorks, Inc., Natick, MA). The calculation time is 2 s for a single‐shot treatment plan and 20 s for a 12–shot treatment plan. It therefore may serve as a secondary verification system for the GammaPlan® calculation at the point of radiosurgery. In the future, we will integrate the algorithm into a Windows based program that can run on any PC. The program will be available at the American Association of Physicists in Medicine (AAPM) software exchange web site (http://aapm.org/resources/software/index.htm).

## References

[1] E. Alexander III , J.S. Loeffler , and L.D. Lunsford , Stereotactic Radiosurgery (McGraw Hill, New York, 1993).

[2] L. Walton , C.K. Bombord , and D. Ramsden , “The Sheffield strereotactic radiosurgery unit: Physical characteristics and principles of operation,” Br. J. Radiol. 60, 897–906 (1987). 3311273 10.1259/0007-1285-60-717-897

[3] A. Wu , G. Lindner , A.H. Maitz , A.M. Kalend , L.D. Lunsford , J.C. Flickinger , and D. Bloomer , “Physics of Gamma Knife Approach on Convergent Beams in Stereotactic Radiosurgery” Int. J. Radiat. Oncol., Biol., Phys. 18, 941–949 (1990). 2182583 10.1016/0360-3016(90)90421-f

[4] D. Kondziolka , K. Dempsey , and L.D. Lunsford , “A comparison between magnetic resonance imaging and computed tomography for stereotactic coordinate determination,” Neurosurgery 30, 402–407 (1992). 1620305 10.1227/00006123-199203000-00015

